# Assessing the Adverse Effects and Safety Concerns Related to Cosmetic and Skincare Products: A Systematic Review

**DOI:** 10.7759/cureus.81759

**Published:** 2025-04-05

**Authors:** Rimaz Sami Alyahya, Maha A AlHasson, Mnahal A Ali Alhsaon

**Affiliations:** 1 Dermatology, Qassim University, Riyadh, SAU; 2 Gynecologist, King Fahad Specialist Hospital, Qassim, SAU; 3 Family Medicine, Department of Public Health, Qassim Health Cluster, Qassim, SAU

**Keywords:** adverse effects, cosmetic safety, personal care products, skincare products, systematic review

## Abstract

Cosmetics and personal care products are essential for personal hygiene and appearance; however, their safety remains a key concern due to associated adverse effects. This systematic review aims to evaluate the adverse effects and safety concerns related to skincare products and to analyze product characteristics and usage patterns for improved consumer safety. The study adhered to the Preferred Reporting Items for Systematic Reviews and Meta-Analyses (PRISMA) guidelines and adopted diverse database searches across PubMed, Medline, Google Scholar, and Scopus from 2014-2024. Only peer-reviewed and cross-sectional studies were included along with a data extraction sheet, and the Critical Appraisal Skills Programme (CASP) tool was utilized for quality assessment. Data synthesis involved descriptive and qualitative analysis to identify common themes and implications. Nine studies were included in the review, comprising 4,569 participants across geographically diverse regions. The common adverse effects reported included acne (36%), redness (27%), itching (19%), and skin irritation (18%). Ingredient analysis identified that fragrances, preservatives, and colorants are commonly related to adverse effects. Usage patterns like frequency and duration of usage were correlated with the likelihood of adverse effects. The review focused on significant adverse effects linked with cosmetic and personal care products, highlighting the necessity for better awareness, clear labels, and strict regulations. Enhanced customer education and following safety protocols are crucial to minimize risks and ensure product safety.

## Introduction and background

Cosmetics and personal care products are integral to modern lifestyles, playing a crucial role in hygiene, appearance, and well-being. Cosmetics are considered a crucial part of today's world in terms of altering the overall appearance and maintaining good shape [1]. As per the European Regulation (EC) No. 1223/2009, cosmetics are defined as substances that are specifically to be used for external body parts or the mucous membranes of the oral cavity with the primary goals of cleansing, beautifying, altering appearance, protecting, or maintaining good condition [2]. Numerous studies have been performed to advocate the efficacy and adverse effects of cosmetics on diverse populations. The use of these products is widespread across all demographics and regions, driven largely by consumer interests in personal appearance and hygiene [1]. An online survey by Cosmetics Europe in 2017 revealed that 71% of respondents considered cosmetics essential in their daily lives, while 72% believed these products enhanced their quality of life [3].

Studies indicate that cosmetics are formulated from a blend of ingredients and must comply with safety standards. Numerous regulations have been designed to ensure the safety and efficacy of skincare products for enhanced consumer safety and to minimize risks. The European Regulation mandates that a cosmetic product should be safe for use under normal or foreseeable conditions. To ensure safety, consumption data, such as frequency, amount, and daily use, are necessary to evaluate exposure levels [4]. These regulations are necessary to ensure the safety and efficacy of consumer risks posed by frequent usage of cosmetic products. This exposure is assessed by dividing the daily product use by the consumer's body weight, resulting in a systemic exposure dose (SED) for each ingredient. This SED is then compared to a No Observable Adverse Effect Level (NOAEL) to calculate the Margin of Safety (MoS) [5]. Early studies in the 2000s provided critical data on cosmetic consumption and exposure across Europe and the United States. However, gaps remain, particularly for products like baby wipes, hair dye, and sunscreen, and for certain populations such as young children and pregnant women [6].

The current literature has majorly focused on addressing this gap. Among them, studies conducted in the 2010s onwards highly focused on the investigation of various populations and product types, offering new insights into cosmetic consumption patterns and exposure. These studies range from large-scale surveys to targeted research on specific subpopulations [7]. European studies highlight that safety assessments were majorly targeting the individual ingredients, but aggregate exposure models have been developed to account for the daily co-use of multiple products, such as the Probabilistic Aggregate Consumer Exposure Model (PACEM) and the Creme Research Institute for Fragrance Materials (RIFM) models. Despite these advancements, concerns persist regarding the safety of cosmetics due to the cumulative effects of their numerous ingredients [8].

Among the variety of cosmetic products, the diverse lists include skin moisturizers, perfumes, lipsticks, nail polishes, facial makeup, and hair care products. While these products can enhance appearance and personal satisfaction, they are not without risks. Adverse reactions, including dermatitis, allergic reactions, and other sensitivities, can occur either immediately or after prolonged use [9]. For instance, a study in Naples reported that 26.5% of women experienced adverse effects from cosmetic products. In Ethiopia, 18.4% of users reported adverse reactions primarily from deodorants and lotions, while a study at Wollo University found that 31.8% of female students experienced issues with lotions and body creams [10].

The global cosmetic market is expanding rapidly, driven by a growing consumer focus on appearance and personal care. Women, in particular, use cosmetics more frequently, often placing higher value on self-image and beauty. This heightened usage, combined with limited awareness of potential risks, underscores the importance of thorough safety evaluations and regulatory oversight. Despite guidelines and safety assessments, the prevalence of adverse effects remains a significant concern [11-19]. Several studies reported that cosmetics acts like poison for our skin which damage slowly but badly [20-24].

The primary aim of this review is to evaluate the adverse effects associated with the use of cosmetics and skin care products, focusing on the safety concerns that arise from both immediate and long-term use. By analyzing current data and identifying gaps in existing research, the study seeks to provide a comprehensive understanding of the risks associated with these products and to recommend strategies for improving consumer safety. The findings will be significant in enhancing regulatory measures, informing consumer practices, and guiding future research in cosmetic safety.

## Review

Methodology

**Figure 1:**
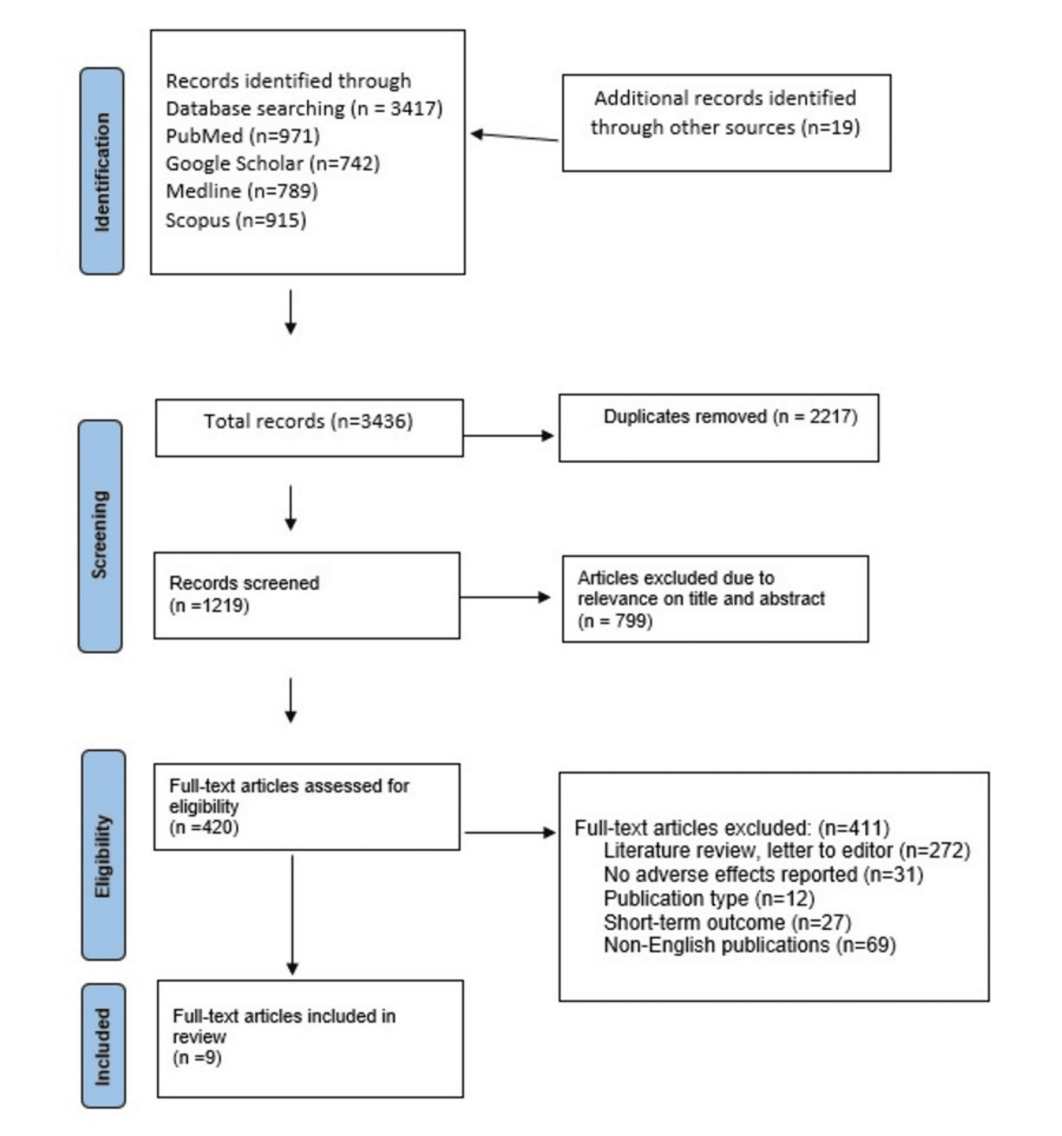
The PRISMA flow chart for the study PRISMA: Preferred Reporting Items for Systematic Reviews and Meta-Analyses

This systematic review, adhering to the Preferred Reporting Items for Systematic Reviews and Meta-Analyses (PRISMA) guidelines, assesses the safety and adverse effects of skincare and cosmetic products, focusing on studies published between 2014 and 2024 (Figure 1). Guided by the PICO (Patient, Intervention, Comparison, Outcome) process - examining reported adverse effects and safety concerns in females using these products - four digital databases (PubMed, Google Scholar, Scopus, and Medline) were searched using key terms, boolean operators, and MeSH terms to ensure comprehensive coverage. Inclusion criteria required cross-sectional, peer-reviewed studies in English focusing on human exposure, while exclusions applied to studies before 2014, non-cross-sectional designs, non-human data, or incomplete findings. A two-stage selection process involved screening titles and abstracts, removing duplicates, and full-text evaluations by two independent reviewers to minimize bias. Data extraction followed a standardized sheet, capturing study type, population characteristics, product types, and exposure parameters (see Appendices). The Critical Appraisal Skills Programme (CASP) tool assessed study quality, ensuring methodological rigor. Descriptive statistics and thematic analysis identified patterns in adverse effects and safety concerns, with findings discussed in relation to consumer safety and regulatory implications, highlighting areas for further research.

Results

Characteristics of the Studies

A total of 3436 records were identified from a diverse literature database, of which 1219 were screened after removing 2217 duplicates. From the screened records, 420 full-text articles were assessed for eligibility. Out of these, 411 articles were excluded for reasons including being literature reviews or letters to the editor (272), lacking reported adverse effects (31), not meeting the publication type criterion (12), focusing on short-term outcomes (27), or being non-English publications (69). Ultimately, nine full-text articles met the inclusion criteria and were included in the review, reflecting a highly selective process to ensure the relevance and quality of the included research.

Study Participants and Baseline Characteristics

Our systematic review encompasses a total of 4,569 participants from nine studies across various locations, including Pakistan (1), Lebanon (1), Sri Lanka (1), Ethiopia (3), Egypt (1), and India (2). The studies predominantly used cross-sectional and descriptive cross-sectional designs. Participants' ages ranged from 15 to 50 years, with a notable concentration in the 18-26 age range, and the majority being females (89%). Commonly used cosmetics included face creams, lipsticks, shampoos, deodorants, and hair dyes, with varying frequencies of use reported, ranging from daily to occasional application. Adverse effects were frequently reported and included acne (36%), redness (27%), itching (19%), and skin irritation (18%), with significant concerns about safety, particularly related to product sharing and the addition of water or saliva to cosmetics. The CASP scores for the studies varied, with most studies scoring between 7 and 9, reflecting generally high methodological quality and rigorous evaluation.

Prevalence and Types of Adverse Effects

Kumari et al. (2023) found that colorants, usually synthetic dyes, are majorly linked to allergic responses in most of the respondents [15]. Meharie et al. (2015) identified that regulatory bodies must enforce strict safety standards to ensure that all products are pretested for safety and efficacy before reaching customers [21]. Three studies identified adverse effects of personal care products, which included skin irritation, allergic reactions, and systemic effects. Nisar et al. (2024) reported skin irritation as the major issue associated with symptoms ranging from mild redness and itching to severe dermatitis [24]. Chahine et al. (2023) found allergic reactions, though less common, that manifested as hives, eczema, and contact dermatitis [6]. Udayanga et al. (2023) analyzed the systemic effects, including respiratory issues and gastrointestinal disturbance, and found them rare but significant in the female population. Gender differences also played a key role - a high prevalence was reported in the female population due to their frequent use of a variety of personal care products.

Safety Concerns and Product Characteristics

Three studies have reported the analysis of ingredients, which revealed that certain ingredients are frequently associated with adverse effects. Among them, the major ingredients were fragrances, preservatives (such as parabens), and colorants. Udayanga et al. (2023) reported that fragrances are commonly known to cause allergic reactions and skin sensitivities. While Dibaba et al. (2014) mentioned that preservatives can provoke dermatitis and other skin irritations [8]. 

Product Usage Patterns

Five studies have found that the type of personal care product and their usage pattern greatly influence the likelihood of causing adverse effects. Among them, two studies found that skincare products, especially those applied recurrently or left on the skin for longer periods of time are more likely to cause reactions compared to rinse-off products like shampoos. Kumari et al. (2023) and Meharie et al. (2015) found that the usage frequency and duration also play a key role - higher frequency or prolonged use of products usually increases the chance of developing adverse effects [21,15]. El Emam et al. (2022) identified that products with complex formulations or multiple active ingredients also increase the risk [9]. The presence of allergens in formulations is a key issue, and products with fewer allergens are linked with a lower incidence of adverse effects.

Risk Factors and Preventive Measures

Three studies identified that individual risk factors can predispose users to adverse effects. Meharie et al. (2015) identified that skin conditions, including eczema or psoriasis, are more prone to develop due to irritation or allergic reactions on the skin. Genetic predisposition also played a key role among a few people with high sensitivity issues to specific cosmetic producers [21]. Paikray et al. (2024) identified that personal care practices like patch-testing new products or using products inconsistently can also influence the onset of adverse effects.

For the mitigation of these skin issues and adverse effects, three studies provided preventive measures, including clear product labeling that includes lists of ingredients and potential allergens. These labels are crucial for user safety. Also, El Emam et al. (2022) identified that ingredient safety guidelines should be followed to limit exposure to known irritants and allergens [9]. Getachew et al. (2018) analyzed that user education on the importance of patch-testing new products and recognizing early signs of adverse reactions are also preventive measures with positive outcomes. 

Discussion

This systematic review aimed to evaluate the prevalence, types of adverse effects, and safety concerns associated with cosmetic use and skincare products among diverse populations. The findings indicate that skin irritation, allergic reactions, and systemic effects are most predominant adverse effects reported in the literature. Among them, skin irritation is a common cause. Allergic reactions usually included hives and eczema, which were highly reported in the studies. Systemic effects included respiratory issues, highlighted by three studies [6,8,11]. In terms of gender differences, the female population was highly vulnerable due to its frequent usage of a variety of products. Safety concerns were linked to ingredients like fragrances, preservatives, and colorants, which led to adverse effects. Individuals with existing skincare issues were more prone to adverse effects. Mitigative measures included patch-testing and strict regulations to improve product safety and minimize associated risks.

In contemporary society, cosmetic products are widely used to enhance physical appearance, yet their use is associated with a range of adverse effects. Our study highlights that 34.4% of participants use cosmetics daily, with 47.7% preferring chemical-based products over 30.9% who opt for Ayurvedic alternatives. Color cosmetics, such as foundations (26.9%), nail paints (23%), and lipsticks (21.5%), are the most frequently used [12]. Similar findings have been reported by Okereke et al. (2015), who found that makeup and personal care products were commonly used, with 24.56% and 22.43% usage rates, respectively [13]. Previous studies consistently show that younger women use cosmetics more frequently than older women, aligning with our results where 190 young women aged 18 to 27 were major users. This trend is often linked to self-care and beautification needs, exacerbated by seasonal and climatic variations affecting skin conditions [12-14].

The study further reveals that women purchase cosmetics based on personal preferences and needs, with most buying color cosmetics monthly and skin/hair care products bi-monthly. This buying pattern reflects the purpose of enhancing appearance and self-esteem, echoing earlier research that women use cosmetics to improve attractiveness and mask imperfections [15]. Notably, an earlier study highlighted that facial and hair cosmetics often contain heavy metals, which can accumulate and cause toxicity [16]. Our findings align with these concerns, as most women reported using chemical-based products, which have been associated with more adverse events compared to Ayurvedic products [17].

Studies have documented various adverse effects of cosmetics [18-22]. Mohiuddin et al. (2019) reported that 36.3% of participants experienced burning and 32.9% had itching due to cosmetics [23]. Lucca et al. (2020) also found that redness (19%), pimples (15%), and itching (13%) were common adverse effects [18]. Our study corroborates these findings, with pimples (19.9%), redness (17.6%), and eye irritation (15.8%) being the prevalent issues. The adverse effects of cosmetics are linked to their source, with local shop purchases often leading to higher risk compared to products from supermarkets. The use of cosmetics from local shops has been associated with increased adverse events, a concern supported by previous research indicating that products from less regulated sources are more likely to cause issues [19-20]. 

The current study also observed that 69.4% of women share cosmetics, which significantly increases the risk of adverse events. This finding is consistent with research by El Emam et al. (2022), which reported a 50% rate of adverse effects among those who shared cosmetics [9]. Furthermore, a study by Udayanga et al. (2023) found that sharing cosmetics contributes to higher risks of infections and other adverse reactions [25]. Our study's results reinforce these concerns, highlighting the need for improved awareness regarding the risks of sharing cosmetics and the importance of adhering to safety measures.

Regarding knowledge and awareness, our findings indicate that many participants are unaware of the full range of adverse effects associated with cosmetics. For instance, 88.7% were unaware that dandruff could be a side effect of hair dyes, a finding consistent with research by Lin et al. (2018), which reported similar gaps in awareness [16]. Studies by Koniecki et al. (2011) [14] and AlRadini et al. (2021) [1] reveal that while many individuals understand the benefits of sunscreen, they are often misinformed about the optimal usage times, reflecting a broader issue of knowledge gaps in skincare practices.

Further studies have shown that the frequency and context of cosmetic use significantly impact adverse reactions. AlRadini et al. (2021) noted that multiple applications of cosmetics could increase the risk of adverse effects due to ingredient interactions [1]. Our study's results align with this, as participants frequently used multiple cosmetic products, potentially heightening their exposure to adverse reactions [22]. Additionally, the usage of cosmetics from unregulated sources, such as non-specialized stores, has been linked to a higher incidence of adverse effects, corroborated by findings from Boome (2018), which emphasize the importance of purchasing from reputable sources [4].

Table 1 presents the risk of bias assessment of the nine studies reviewed in this article. 

**Table 1:** Risk of bias assessment based on the Joanna Briggs Institute (JBI) checklist for cross-sectional studies. Low Risk of Bias: Studies that met all criteria and had a good response rate; Moderate Risk of Bias: Studies that lacked response rate details or had missing sample selection criteria; High Risk of Bias: If there were major concerns in sampling, data collection, or validity of measurements.

Study	Appropriate Sample Frame	Adequate Sample Size	Appropriate Sampling	Data Collection Methods	Valid Measurements	Response Rate	Overall Risk of Bias	References
Nisar et al. 2024	Yes	Yes	Yes	Yes	Yes	Moderate (missing details on response rate)	Low	[24]
Chahine et al. 2023	Yes	Yes	Yes	Yes	Yes	High (low response rate not mentioned)	Moderate	[6]
Udayanga et al. 2024	Yes	Yes	Yes	Yes	Yes	Low	Low	[25]
Dibaba et al. 2013	Yes	Yes	Yes	Yes	Yes	Moderate	Low	[8]
Kumari et al. 2023	Yes	Yes	Yes	Yes	Yes	Low	Low	[15]
El Emam et al. 2022	Yes	Yes	Yes	Yes	Yes	Low	Low	[9]
Meharie et al. 2014	Yes	Yes	Yes	Yes	Yes	Moderate	Moderate	[21]
Getachew et al. 2018	Yes	Yes	Yes	Yes	Yes	Moderate	Moderate	[11]
Paikray et al. 2024	Yes	Yes	Yes	Yes	Yes	Low	Low	[26]

Overall, this study's results provide valuable insights into cosmetic use patterns, adverse effects, and associated knowledge gaps among women. By comparing our findings with existing research, it is evident that while cosmetic use is prevalent and often aimed at enhancing appearance and self-esteem, there are significant concerns regarding the safety and awareness of these products. Increased education on the risks associated with cosmetic use and proper purchasing practices is essential to mitigate adverse effects and ensure safer cosmetic practices.

The present study had several limitations. Firstly, the use of self-administered surveys may have introduced biases, such as social desirability and recollection biases, potentially affecting the accuracy of the data. Additionally, the estimates of adverse events were based on self-reports, which could lead to underestimation of the true frequency of these events. Moreover, while the study covered diverse regions, the findings may not be fully generalizable to other populations or areas. The study’s region-specific nature and reliance on self-reported data should be considered when interpreting the results.

## Conclusions

In conclusion, this systematic review underscores the significant prevalence of adverse effects associated with personal care and cosmetic products, including skin irritation, allergic reactions, and systemic effects. The findings highlight that certain product ingredients, such as fragrances, preservatives, and colorants, are frequently linked to these adverse effects. Additionally, the frequency and type of product use, along with individual risk factors, play crucial roles in determining the likelihood of adverse reactions. The review also emphasizes the need for better awareness, clearer product labeling, and stringent regulatory measures to mitigate risks and enhance user safety. This study has several limitations that should be considered. Selection bias may affect the generalizability of findings, as the sample might not fully represent broader populations. Self-reported data introduce recall and response biases, potentially impacting accuracy. Additionally, the cross-sectional design prevents causal inferences. Future research should address these limitations by using randomized sampling to enhance representativeness, conducting longitudinal studies to capture changes over time, and incorporating objective measures to minimize biases. Expanding studies to diverse populations and multi-center settings will further improve the applicability of findings across different contexts.

## Appendices

**Table 2:** Data extraction sheet of selected studies

Author name and year of publication	Study design	Location	Participant characteristics	Commonly Used Cosmetic Products	Frequency of Use	Adverse Effects Reported	Safety Concerns	Main Findings	CASP Score
Nisar et al. 2024 [24]	Cross-sectional study	Pakistan	- Total Number: 392 women - Age Range: 18 to 50 years; Mean Age: 34 years -Age Distribution: 57.1% (18-27 years), 35.2% (28-39 years), 7.7% (40-50 years) - Marital Status: 73.7% single, 26.3% married -Educational Status: 86.2% university, 7.6% college, 6.1% school	-Color Cosmetics: Foundations (26.9%), Nail Polish (23%), Lipstick (21.5%), Eyeliner (16.4%), Mascara (11.5%) - Skin Care Products: Face Wash (39.5%), Moisturizers (26.5%), Sunscreens (23.2%), Body Wash (7.7%), Anti-aging Creams (3.1%) - Hair Care Products: Shampoos (75.5%), Conditioners (17.1%), Hair Colors (4.8%), Styling Gels/Sprays (1.8%), Protein Packs (0.8%) - Fragrances: Deodorant Sprays (46.7%), Perfumes (36.7%), Deodorant Sticks (16.6%)	- Color Cosmetics: Occasionally (53.3%), Daily (34.4%), Rarely (9.4%), Never (2.8%) - Skin Care Products: Daily (97.4%), Occasionally (2.0%), Rarely (0.5%) - Hair Care Products: Occasionally (6.4%), Rarely (2.6%), Daily (1.0%) - Fragrances: Occasionally (48.7%), Rarely (34.9%), Daily (12.5%), Never (3.8%)	- Pimples (19.9%), Redness (17.6%), Eye Irritation (15.8%), Rash (13.8%), Pigmentation (7.7%), Eczema (7.1%), Burning (5.1%), Nausea (5.1%), Itching (4.8%), Dizziness (3.1%)	- Sharing Cosmetics: 69.4% shared cosmetics, 30.6% did not - Reading Expiry Date: 73.7% read expiry dates, 26.3% did not - Source of Purchase: Local Shops (62%), Supermarkets (21.7%), Drug Retail Outlets (13%), Online Sources (3.3%)	- Preference for chemical-based cosmetics (47.7%) and Ayurvedic products (30.9%) - Common adverse events include pimples, redness, and eye discomfort - High proportion (51.3%) agreed cosmetics help achieve a whiter complexion - Importance of encouraging cosmetovigilance and awareness campaigns among cosmetic product sellers and users	9
Chahine et al. 2023 [6]	Cross-sectional survey	Lebanon	Total Number: 1,051 Age: Majority (70.8%) between 18 and 20 years Sex: Female Background Data: Majority single (87.4%), high school degree (62.4%), unemployed (76.7%)	Cleansers, sunscreens, hair dye, waterproof mascara, color-depositing shampoo	- 1–2 cosmetics per day: 44.7% - Twice daily use: 59%	Skin allergies (perfume), dandruff (hair dye), skin irritation and sensitivity, yellow discoloration (nail polish)	Concerns regarding inappropriate use causing rashes, skin darkening, wrinkles; use of hair dye during pregnancy; potential endocrine system harm	- Knowledge Mean Score: 7.54 ± 2.7 (range 0–14) - 62.6% classified as knowledgeable - Positive attitude towards cosmetics - Significant associations with area of residency and monthly income - Need for increased awareness on acute and chronic side effects of cosmetics	8
Udayanga et al. 2024 [25]	Descriptive cross-sectional study	Sri Lanka	Total Number: 421 undergraduates Age: 20-23 years (55.1%), 24-26 years (41.3%) Sex: 78.4% female, 21.6% male Ethnicity: Sinhala (71.3%), Muslim (17.6%), Tamil (10.7%) Residency: Semi-urban (51.1%), Rural (29.7%) Academic Fields: Technology (36.6%), Agriculture (20.2%), Biological Sciences (19.7%), Commerce and Management (11.2%) Academic Year: First year (32.3%), Fourth year (17.8%)	Perfumes (65.6%), Face cream (63.2%), Body lotion/hand cream (60.6%)	Once a day (42.3%), Twice a day (32.8%)	Skin dryness (24%), Acne (21%), Allergies (20.5%), Rashes (19.8%)	Limited knowledge (47.5% low knowledge), Significant adverse health effects (75.3%)	- High prevalence of cosmetic use and adverse effects among undergraduates - Significant factors: gender, academic year, knowledge on cosmetics, expenditure, advice source - Good practices during purchase and application noted, but many had low knowledge on cosmetics	9
Dibaba et al. 2013 [8]	Cross-sectional study	Ethiopia	Total Number: 742 (response rate: 93.1%) Age Range: 19 to 24 years Mean Age: 21.4 years Sex: Female Ethnic Groups: Tigre (33.1%), Oromo (28.6%), Amhara (19.9%), Others (18.4%) Religion: Orthodox Christians (47.7%), Protestants (32.4%), Muslims (18.6%) Monthly Income: 500 birr and above (50.3%), less than 500 birr (49.7%)	Body lotion (76.0%), Deodorants (74.0%), Hair cosmetics (51.3%)	Mean number of products used per day: 2.78 Median number of products used per day: 2 Percentage using >5 products per day: 4.8%	Allergic reactions, inflammation, hair loss, discoloration, brittle hair, sores on skin and face, bleeding on scalp, stinging, darkening of armpits Affected Body Parts: Face and hair (42.9% each) Percentage Reporting Adverse Effects: 18.4%	Consulted Health Professionals: 11.1% Testing for Allergies: 24.8% Reading Labels: 65.8% Sharing Cosmetics: 48.6% Adding Water/Saliva to Cosmetics: 17.6%	Majority of participants used cosmetics; significant proportion experienced adverse reactions Determinants: Increased utilization with higher income; more adverse reactions with higher number of products used per day, sources of cosmetics, and sharing of cosmetics Recommendations: Awareness on rational cosmetics use to decrease adverse reactions	9
Kumari et al. 2023 [15]	Cross-sectional study	India	- Total Number: 400 - Age Range: 15 to 50 years - Sex: 160 males (40%), 240 females (60%) - Background Data: 76.5% urban, 51% graduate or higher education, 12.2% with food/drug allergy	- Makeup: 35.9% - Personal Care Products: 28% - Skin Care Products: 46%, Hair Care Products: 28.1%, Personal Care Products: 17.3%	- 1–2 cosmetics per day: 54.7% - Twice daily use: 49%	- Prevalence of Adverse Events: 33% - Most Common Adverse Effects: Itching (58), Redness (59), Pimples (42) - Management: Cessation of use (69.7%), Physician consultation (22.7%), Medication (20.4%)	- Performing Allergy Test: Those who did not perform an allergy test were more likely to experience adverse events (AOR 1.99; 95% CI 1.04–3.80) - Changing Cosmetic Brands Frequently: Lower risk of adverse events (AOR 0.50; 95% CI 0.32–0.78)	- High prevalence of adverse events related to cosmetics use. - Need for increased awareness about rational cosmetic use and establishment of a cosmetovigilance center.	8
El Emam et al. 2022 [9]	Cross-sectional study	Egypt	- Total Number: 790 female university students - Age: Mean age 17.7 years ± 1.9 SD; Range: 6-22 years - Sex: Female - Background Data: Students from medical, practical, and theoretical faculties; stratified cluster sampling used	- Soap (98%), Toothpaste (96.1%) - Shampoo (88.7%), Hair Cream (86%), Lipstick (75.5%) - Hand Lotion (77.9%), Shower Gel (77.7%), Body Lotion (74.9%)	- Soap: More than 3 times a day (69%) - Toothpaste: Twice per day (42.3%) - Eye Makeup, Lipstick, Face Mask, Shower Gel: Once per day (~50%) - Hair Dyes and Contact Lenses: Least frequent use	- Acne (56.6%), Redness (34.7%), Darkening of the Armpits (30.0%), Itching (26.3%), Eye Inflammation (28.8%) - Hair Breakage (20.0%), Soreness (14.6%), Facial Discoloration (13.9%), Skin Burns (10.7%) - Affected Areas: Face (63.2%), Eyes (38.6%), Armpits (29.7%), Lips (24.7%)	- 21.9% checked if cosmetics were tested on animals - 46.7% tested cosmetics for allergic reactions - 94.4% removed makeup before bed; 88.7% read expiry date; 75.3% read safety warnings - 49.3% added water/agents to cosmetics; 40.2% shared cosmetics with others	- High prevalence (87.5%) of cosmetic use among university female students - Significant predictors: Age, mother's education, residence, family size, income - Common reasons for use: Beautification (60.8%), Entertainment (54.7%), Body Protection (41.4%) - High self-reported adverse events (84.7%); frequent adverse effects include acne, redness, and itching - Need for educational interventions to raise awareness about potential harmful effects of prolonged use	9
Meharie et al. 2014 [21]	Cross-sectional study	Ethiopia	227 (after excluding 7 incomplete responses) - Age Range: 19 to 26 years - Mean Age: 22.5 years - Sex: Female - Marital Status: 93.2% unmarried, 6.8% married - Ethnicity: 49.1% Amhara, 19.5% Tigrea, 18.6% Oromo, 12.7% other - Religion: 64.1% Orthodox Christian, 20% Protestant, 15.5% Muslim - Monthly Income: 52.7% ≥500 birr, 47.3% <500 birr	- Lotions 89.7% - Hair Cosmetics 88.8% - Deodorants 62.6%	More than 2 cosmetics per day 55.2% - Mean Number of Cosmetics Used Per Day 3.34	Proportion of Users Reporting Adverse Effects: 31.8% - Common Adverse Effects: Itching (79.4%), Acne (77.9%) - Types of Products Causing Adverse Effects: Lotions (75%), Body creams (72.1%)	Cosmetics Sharing Associated with increased adverse effects (OR=1.950, 95% CI=1.606−6.105) - Adding Water/Saliva Associated with increased adverse effects (OR=2.365, 95% CI=1.052-5.885) - Traditional Cosmetics Associated with increased adverse effects (OR=2.127, 95% CI=1.334-6.036) - Source of Cosmetics Buying from drug retail outlets associated with fewer adverse effects (OR=1.740, 95% CI=1.088−2.954)	High Prevalence of Cosmetic Use 97.3% of participants used cosmetics - Cosmetics Usage Patterns Common products include lotions and hair cosmetics; use of more than five cosmetics per day associated with higher adverse effects - Cosmetics Utilization Practices Use of traditional cosmetics, adding water/saliva, and sharing cosmetics are prevalent; limited label reading and allergy testing	7
Paikray et al. 2024 [26]	Observational study	India	Total Number: 120 patients - Age Range: Above 18 years - Gender: Both males and females (females outnumbered males) - Demographic Data: Majority were married, 48.3% aged 18-29 years, most had passed class 10	- Face care products (n=144) - Make-up care products (n=126) - Personal care products, perfumes, deodorants, nail care products	- 1–2 cosmetics per day: 65.7% - Twice daily use: 35%	- Acne, form eruption, Scaling, Erythema, Rashes - Burning sensation, Itching, Hair fall Pigmentation Dryness of skin, Vesicular lesions, Perioral dermatitis, Plaque	Face care products were most frequently associated with adverse effects - Acne was the most common ACR reported	Majority of ACRs were caused by facial care products (51.2%) - Acne was the most frequently occurring ACR - Causality assessment using COLIPA method: 49.4% likely; PLM method: 59% probable - Increased awareness and reporting encouraged	7
Getachew et al. 2018 [11]	Cross-sectional study	Ethiopia	426 female employees (academic, administrative, and health professionals) from Jimma University. Sampling: Stratified random sampling. Majority aged 25-29 years; 68.5% had education beyond secondary school. Income: 71.9% earned below 3000 ETB/month.	80.1% used cosmetics. Most common products: toothpaste, lotion, lipstick, eye makeup.	79.4% used cosmetics daily. 61.9% used traditional cosmetics.	19.0% experienced adverse events (e.g., hair breakage, body rash, itching). Commonly affected areas: face (36.8%), hair (12.3%). Main causes: lotions, hair cosmetics. Resolution: 59.6% stopped using the suspect product, 14.0% sought medical consultation.	Sharing Cosmetics: 57.6% Adding Water/Saliva to Cosmetics: 15.6%	A significant proportion of female employees experienced adverse effects from cosmetic use. Increased awareness and cautious use of cosmetics are recommended to minimize adverse events.	8
